# Fulminant Non-occlusive Mesenteric Ischemia After Head Trauma: Report of Two Cases

**DOI:** 10.7759/cureus.61227

**Published:** 2024-05-28

**Authors:** Shoko M Yamada, Yusuke Tomita, Naotaka Iwamoto, Mikiko Takahashi

**Affiliations:** 1 Neurosurgery, Teikyo University Mizonokuchi Hospital, Kawasaki, JPN; 2 Diagnostic Pathology, Teikyo University Mizonokuchi Hospital, Kawasaki, JPN

**Keywords:** acute pancreatitis, diarrhea, hyperglycemia, head trauma, non-occlusive mesenteric ischemia

## Abstract

There have been no case reports of non-occlusive mesenteric ischemia (NOMI) following head trauma. Our two patients with non-surgical traumatic intracerebral hemorrhage succumbed to NOMI one week after the injury. Both were women over age 80 years and were clinically improving before NOMI occurred. One patient had been eating since admission, while the other had not, which prompted the initiation of enteral nutrition on day 5. The patients shared many characteristics: 1) over age 80 years; 2) minor brain contusion; 3) constipation for a week; 4) minimal abdominal symptoms; 5) rapidly developing leukocytosis, hyperglycemia, hypernatremia, and elevated blood urea nitrogen; 6) massive diarrhea with a small amount of blood on the same day that laboratory data became abnormal; and 7) fever and shock developed shortly after diarrhea appeared. Because of the fulminant worsening of the condition, shock status, and old age, surgical intervention was considered high risk and not performed in either patient. In retrospect, if NOMI had been diagnosed earlier when the acute pancreatitis-like symptoms began, surgical intervention may have saved their lives. Clinicians should be aware that NOMI can occur after relatively minor head trauma, which can cause death if the diagnosis is delayed.

## Introduction

Non-occlusive mesenteric ischemia (NOMI) occurs without occlusion of the mesenteric arteries, accounting for 20%-30% of all acute mesenteric ischemia cases with an extremely high mortality rate [1-3]. As risk factors of NOMI, followings are included: older than 50 years with a history of ischemic heart disease [1], atherosclerosis [4], dialysis [5,6], diabetes mellitus [7], and enteral nutrition (EN) [8,9]. However, it may be difficult to clarify risk factors because this disorder frequently occurs in critically ill patients [10]. The cause of NOMI is insufficient blood supply to the intestine, which may be triggered by major surgery [7], severe hypotension [10], various types of shock [1], mesenteric arterial vasoconstriction due to drug interaction [1,3], and successful resuscitation from cardiac arrest [11].

Abdominal computed tomography (CT) is essential for the diagnosis of NOMI, and hepatic portal vein gas and thickening of the small intestinal wall are important findings that suggest bowel necrosis [12]. Recently, the diagnosis of NOMI is more definitive on multi-detector CT (MDCT) and CT angiography by observing the diameter of superior mesenteric artery, celiac trunk, and superior mesenteric vein [3,13]. The term “fulminant NOMI” is used to describe a case of sudden onset and rapid worsening of symptoms that resulted in shock and death even after emergency diagnosis and treatment [14].

To the best of our knowledge, NOMI has not been previously reported after head trauma. The two cases in this report died of fulminant NOMI even though they had mild head trauma with good general condition. Therefore, we report here rare cases of fatal NOMI after a nonfatal head injury.

## Case presentation

This is a case series of two cases, experienced at Teikyo University Mizoguchi Hospital in February 2022 and January 2023. Consent was obtained in the form of written signatures from the families of the two cases.

Case 1

**Figure 1:**
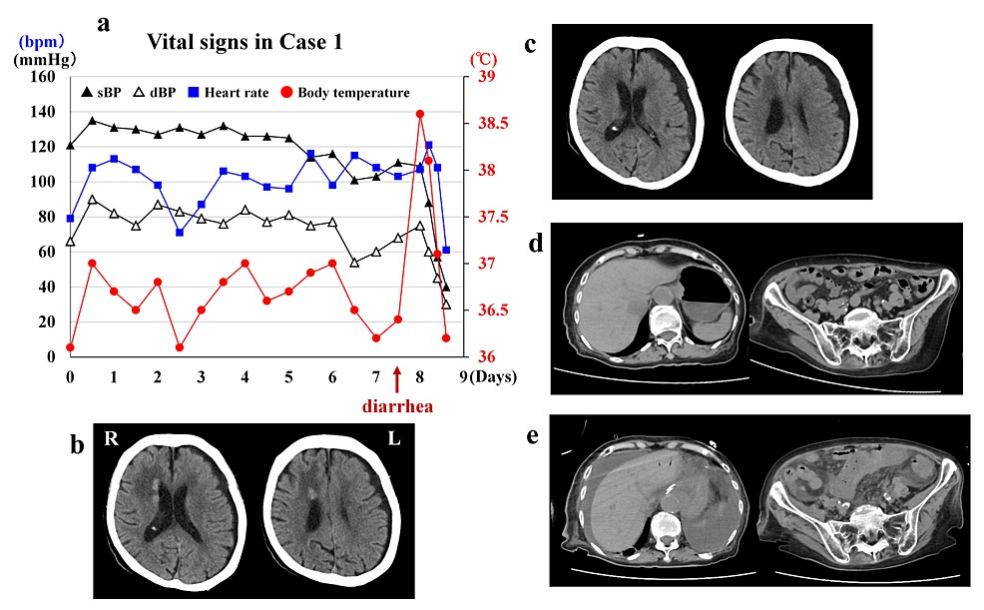
Case 1 (a) Vital signs over time: Until day 6, the patient’s vital signs were stable. Triggered by diarrhea at day 7, her body temperature suddenly became high and subsequently went into shock status. (b) Head CT on admission: High-density areas are identified at the subependymal area along the right lateral ventricle with low density areas around the lesions. Thin subdural hygroma appears in the left side. (c) Head CT on day 2: The high-density areas along the right ventricle disappeared but left subdural space enlarges compressing the left hemisphere. (d) Abdominal CT on day 5: Apart from moderate amount of air in the stomach, there is no intestinal edema, stool accumulation, or ileus. (e) Abdominal CT on day 7: Compared with the CT on day 5, massive ascites is remarkable. The hepatic portal vein gas and marked edema of the small bowel are also identified.

An 86-year-old woman with a history of hypertension struck her head on the living room floor during a family visit to her home. On hospital arrival, her height and weight were 152 cm and 48.5 kg, respectively. She was amnestic to the event and disoriented. Glasgow Coma Scale (GCS) score was 14 (E4V4M6). Her vital signs on arrival (day 0) and throughout hospitalization are shown in Figure 1a. Head CT demonstrated a left subdural hygroma and subependymal areas of high signal density along the right lateral ventricle. The patient was admitted for observation. Follow-up CT on day 2 showed enlargement of the left subdural hygroma and blurring of the high-density areas. On day 5, she complained of decreased appetite and no bowel movements since admission. Oral intake had previously been adequate. Abdominal CT was unremarkable. Laboratory data on day 7 showed leukocytosis (26.6 × 10^3^/mL), high C-reactive protein (CRP) concentration (11.6 mg/dL), high blood urea nitrogen (BUN) concentration (62.0 mg/dL), hypernatremia (153 mmol/L) and hyperglycemia (554 mg/dL) (Table 1). Her vital signs remained stable. Acute pancreatitis was suspected, but she had no abdominal or back pain, and concentrations of amylase and lipase levels were normal (Table 1). An intravenous insulin drip was initiated. That evening (day 7), after defecating watery stools with a tiny amount of blood, the patient’s level of consciousness deteriorated rapidly. On examination, her GCS score was 6 (E2V1M3) and she grimaced when her abdomen was palpated. Abdominal CT demonstrated massive ascites, gas in the hepatic portal venous system, and edema throughout the small intestine, which strongly suggested NOMI. Her family declined emergency surgery because of her advanced age. Palliative treatment and supportive care were administered. On day 8, her serum glucose and sodium concentrations normalized to 125 mg/dL and 144 mmol/L, respectively; her leukocytosis had worsened (70.4 × 10^3^/mL), however. The patient died later that day. An autopsy was not performed (Figures 1a-1e).

**Table 1:** Laboratory data in case 1 WBC: white blood cell, Hb: hemoglobin, Hct: hematocrit, Plt: platelets, CRP: C-reactive protein, BUN: blood urea nitrogen, Cr: creatinine, Na: sodium. K: potassium, Cl: chloride, TP: total protein, CK: creatine kinase, AST: aspartate aminotransferase, ALT: alanine aminotransferase, g-GT: γ-glutamyl transpeptidase, PT-INR: prothrombin time-international normalized ratio, Fib: fibrinogen

	Day 0	2	5	7 (AM 7:00)	7 (PM 2:00)	8
WBC (3.3~8.6 x10^3^/mL)	6.3	7.7	7.8	26.6	48.4	70.4
Hb (11.6~14.8 g/dL)	12.4	11.4	11.5	11.7	11.5	13.1
Hct (35.1~44.4 %)	39.5	38.1	37.4	38.14	36.0	45.4
Plt (15.8~34.8 x10^4^/mL)	17.5	19.2	21.4	19.5	9.5	6.3
CRP (0~0.14 mg/dL)	3.51	8.69	5.61	11.6	10.2	10.6
BUN (8~20 mg/dL)	38.5	26.5	24.5	62.0	61.2	61.3
Cr (0.46~0.79 mg/dL)	1.23	1.09	1.0	1.96	2.05	2.38
Na (138~145mmol/L)	140	144	149	153	146	144
K (3.6~4.8 mmol/L)	3.7	3.7	3.2	3.9	3.3	4.5
Cl (101~108 mmol/L)	108	106	111	119	109	108
TP (6.6~8.1 g/dL)	6.8	6.62	5.94	4.76	3.98	4.32
Albumin (4.1~5.1 g/dL)	3.2	2.8	2.1	2.4	1.77	2.0
CK (41~153 U/L)	99	161	88	37		37
AST (13~30 U/L)	27	17	13	16		203
ALT (7~23 U/L)	18	14	15	8		88
g-GT (9~32 U/L)	35	32	26	21		24
Glucose (mg/dL)	119	106	135	554	225	125
PT-INR	1.39	0.89	1.11			3.5
Fib (170~410 mg/dL)				572		383
D-dimer (mg/mL)	2.6			6.9		7.2
Amylase (44~132 U/L)	45			71		
Lipase (13~55 U/L)	26			32		
HbA1c (≤5.5%)	5.8					

Case 2

**Figure 2:**
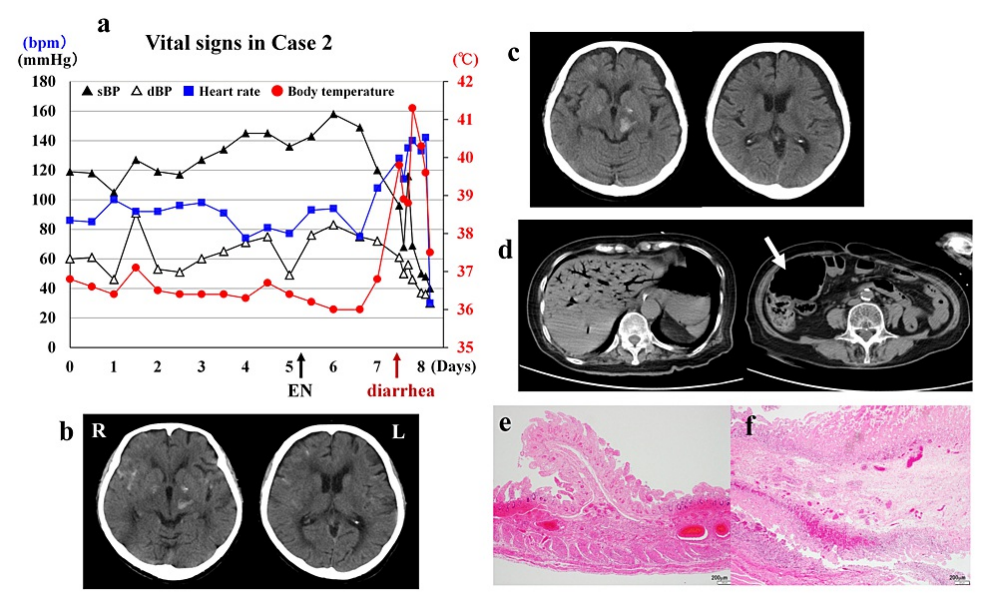
Case 2 (a) Vital signs over time: EN was initiated from day 5. Soon after the diarrhea at day 7, the patient demonstrated extremely high body temperature followed by irreversible rapid drop of blood pressure. (b) Head CT on admission: Traumatic SAH was identified at the right sylvian fissure and sulci in the right frontal, and small intraparenchymal hemorrhage in the left cerebral peduncle. (c) Head CT at day 1: SAH in the right sylvian fissure was washed out and the hemorrhage in the left cerebral peduncle enlarged. Thin subdural hygroma appears at the left side. (d) Abdominal CT at day 7: The CT demonstrated multiple dendritic hepatic portal venous gas, and the ascending colon is markedly enlarged due to gas retention (white arrow). But no obvious necrosis of the small intestine can be clearly recognized. (e) The mucosa of the intestinal tract is in a standing necrotic state (epithelial necrosis with cell denucleation, leaving the framework of the glandular ducts), with intramucosal hemorrhage and dilated blood vessels (hematoxylin and eosin (HE), 40x). (f) The subepithelial mucosal lamina propria is edematous, with a small neutrophilic infiltrate (HE, 40x).

An 83-year-old woman with a history of hypertension and hyperlipidemia presented to the hospital complaining of headache and nausea after sustaining a head injury. Her height and weight were 145 cm and 49.0 kg, respectively. Her vital signs on arrival and throughout hospitalization are shown in Figure 2a. Examination was notable for a GCS score of 14 (E4V4M6) and weakness of the right upper and lower limbs (3+/5 on manual muscle testing (MMT)). Head CT revealed traumatic subarachnoid hemorrhage in the right Sylvian fissure and frontal sulci as well as a small hemorrhage in the left cerebral peduncle. The patient was admitted for observation (day 0). A few hours later, her weakness worsened to MMT 2/5. Repeat CT showed enlargement of the left cerebral peduncle hemorrhage and left subdural hygroma. Because of ongoing nausea and poor oral intake, intravenous fluids were initiated. On day 4, her weakness improved to MMT 4+/5, but dysphagia was observed. A nasogastric tube was inserted to initiate EN on day 5, as bowel sounds were auscultated. The intravenous fluids were also continued. Vital signs were stable until day 6. In the morning of day 7, laboratory data demonstrated leukocytosis (15.0 × 10^3^/mL), hypernatremia (151 mmol/L), increased BUN concentration (47.7 mg/dL) and hyperglycemia (734 mg/dL) (Table 2). Acute pancreatitis was ruled out because of no abdominal pain and normal concentrations of amylase and lipase (Table 2). Insulin was added to her intravenous fluids and EN was continued. In the afternoon, the patient complained of abdominal pain one hour after an EN feeding had completed; she also defecated a large volume of watery stools with a small amount of blood. Thirty minutes later, her body temperature increased to 39.8°C, blood pressure fell to 96/61 mmHg, and her level of consciousness deteriorated (GCS 5: E1V1M3). The glucose concentration had decreased to 46l mg/dL but her hypernatremia and elevated blood urea nitrogen concentration had worsened (Table 2). Head CT showed no new lesions. Abdominal CT demonstrated dendritic hepatic portal venous gas, dilatation of the ileocecal region and marked enlargement of the ascending colon; no obvious necrosis of the small intestine was noted. Despite administration of albumin and catecholamines, the patient’s blood pressure dropped to 67/48 mmHg. Her level of consciousness never recovered, and body temperature increased further to 41.4°C. She died early the next morning (day 8). The family consented to an autopsy, which revealed 500 mL of bloody ascites and necrosis throughout the small intestine. In the terminal portion of the ileum and the ileocecal region, intestinal wall thinning was observed. Microscopic examination findings in the small intestine are presented in Figures 2f-2i. No obstruction of the mesenteric vessels was found, leading to a diagnosis of NOMI (Figures 2a-2f).

**Table 2:** Laboratory data in case 2

	Day 0	3	5	7 (AM7:00)	7 (PM4:00)	8
WBC (3.3~8.6 x10^3^/mL)	8.0	12.8	8.1	15.0	4.2	7.3
Hb (11.6~14.8 g/dL)	13.3	13.1	11.8	14.5	14.9	16.2
Hct (35.1~44.4 %)	36.8	38.4	34.5	41.7	42.4	45.0
Plt (15.8~34.8 x10^4^/mL)	23.2	22.4	20.1	24.7	11.8	9.6
CRP (0~0.14 mg/dL)	0.01	1.49	1.51	6.44	6.17	7.94
BUN (8~20 mg/dL)	13.0	17.8	28.9	47.7	67.9	74.2
Cr (0.46~0.79 mg/dL)	0.52	0.43	0.61	0.96	1.66	2.49
Na (138~145mmol/L)	121	137	136	151	152	154
K (3.6~4.8 mmol/L)	4.2	3.1	2.6	3.1	3.0	4.0
Cl (101~108 mmol/L)	87	99	100	108	117	120
TP (6.6~8.1 g/dL)	7.2	7.2	7.0	6.2	6.5	4.7
Albumin (4.1~5.1 g/dL)	4.2	4.3	3.8	3.2	3.3	2.4
CK (41~153 U/L)	104	84	43	27	442	1108
AST (13~30 U/L)	32	24	34	23	103	578
ALT (7~23 U/L)	18	11	24	27	35	223
g-GT (9~32 U/L)	16	19	20	26	21	22
Glucose (mg/dL)	146	203	153	734	461	268
PT-INR	0.98	0.89		1.13		1.73
Fib (170~410 mg/dL)				572		466
D-dimer (mg/mL)	7.5			4.8		7.3
Amylase (44~132 U/L)	38			47		118
Lipase (13~55 U/L)	18			21		47
HbA1c (≤5.5%)	6.1					

## Discussion

A critically ill state has been considered a precondition for developing NOMI [2,10]. The possibility that EN-induced NOMI in Case 2 cannot be ruled out. However, all the reported NOMI cases induced by EN were in poor general condition [8,9]. In contrast, our two patients were not in critical condition and NOMI was not foreseeable. Furthermore, minimal abdominal symptoms before NOMI onset may have contributed to a delay in diagnosis. Even though the NOMI was fulminant, had it been diagnosed early, surgical intervention may have saved their lives.

Our patients shared many characteristics: 1) over age 80 years; 2) minor traumatic intracerebral hemorrhage; 3) constipation for a week; 4) minimal abdominal symptoms; 5) rapidly progressing leukocytosis, hyperglycemia, and hypernatremia; 6) massive diarrhea with a small amount of blood on the same day that laboratory data became abnormal; 7) acute fever and shock developed shortly after diarrhea appeared. The mortality in NOMI increases with age and is highest in octogenarians without gender difference [2]. There are no previous reports of NOMI developing after head trauma, but the authors believe that NOMI would not have occurred in our patients had the head injuries not occurred, and that the head trauma and consequent cerebral hemorrhage indirectly resulted in NOMI. Constipation itself is not associated with mesenteric ischemia, but bloody diarrhea is [15,16]. Our patients complained of little abdominal pain. Nonetheless, the disease rapidly progressed to such a fulminant stage that no aggressive treatment could be applied. The rapid onset of leukocytosis, hyperglycemia, hypernatremia, elevated BUN concentration, and fever were clear signs of a clinical problem. Hyperglycemia appears to be a rare complication of NOMI and it has only been reported in one other case [17]. However, it is probably not a coincidence that our patients, who did not have a history of diabetes mellitus, demonstrated rapid elevation of serum glucose concentration. The concomitance of pancreatic hypo-function and decreased insulin secretion in NOMI may be explained by the dysfunction of the celiac plexus to the pancreas due to the hypo-perfusion of the proximal portion of the superior mesenteric artery [1]. Deteriorated consciousness could be the early sign of sepsis [18], and the presence of hepatic portal vein gas on abdominal CT suggests that intestinal bacteria have entered the bloodstream. High fever and rapid drop in blood pressure after the development of bloody diarrhea indicated septic shock from intestinal necrosis. At this point, surgical intervention in a patient over 80 years of age would likely have resulted in death. In retrospect, if we had suspected NOMI and made the diagnosis earlier, surgery could have been performed before the shock occurred. When acute pancreatitis-like signs such as leukocytosis, hyperglycemia, hypernatremia, and elevated BUN are observed in an older patient after a head injury, NOMI should be suspected in addition to acute pancreatitis, and MDCT or CT angiography is recommended for early detection of NOMI and for determining the indication for emergency surgical bowel resection [3,13]. Clinicians should be aware that NOMI can occur after head trauma, which can be fatal if not diagnosed promptly.

## Conclusions

Generally, NOMI is a complication in critically ill patients. However, as shown in this report, NOMI can also occur in mild head injury cases admitted for observation, and the most common cause of death due to NOMI is delayed diagnosis. Both our cases were over 80 years old, demonstrated rapidly progressing leukocytosis, hyperglycemia, hypernatremia, and dehydration half a day prior to becoming shock condition, and had massive diarrhea with a small amount of blood a few hours before reaching the hypotensive shock status.

Considering the time from the onset of these abnormalities to the shock status, it is extremely important that NOMI should be kept in mind and adequate emergent examinations should be performed when acute pancreatitis-like data (leukocytosis, hyperglycemia, hypernatremia, and high BUN value) are identified in a patient following head trauma especially in elderly.

## References

[1] Trompeter M, Brazda T, Remy CT, Vestring T, Reimer P (2002). Non-occlusive mesenteric ischemia: etiology, diagnosis, and interventional therapy. Eur Radiol.

[2] Acosta S, Ogren M, Sternby NH, Bergqvist D, Björck M (2006). Fatal nonocclusive mesenteric ischaemia: population-based incidence and risk factors. J Intern Med.

[3] Topolsky A, Pantet O, Liaudet L, Sempoux C, Denys A, Knebel JF, Schmidt S (2023). MDCT-findings in patients with non-occlusive mesenteric ischemia (NOMI): influence of vasoconstrictor agents. Eur Radiol.

[4] Kawada T (2021). Atherosclerosis is a risk factor of mortality in patients with non-occlusive mesenteric ischemia. Eur J Radiol.

[5] John AS, Tuerff SD, Kerstein MD (2000). Nonocclusive mesenteric infarction in hemodialysis patients. J Am Coll Surg.

[6] Archodovassilis F, Lagoudiannakis EE, Tsekouras DK (2007). Nonocclusive mesenteric ischemia: a lethal complication in peritoneal dialysis patients. Perit Dial Int.

[7] Klotz S, Vestring T, Rötker J, Schmidt C, Scheld HH, Schmid C (2001). Diagnosis and treatment of nonocclusive mesenteric ischemia after open heart surgery. Ann Thorac Surg.

[8] Iapichino G, Radrizzani D, Zanello M, Tetamo R, Buono S (2022). Enteral nutrition and acute mesenteric ischemia. Intensive Care Med.

[9] Ruiz NC, Kamel AY, Shoulders BR, Rosenthal MD, Murray-Casanova IM, Brakenridge SC, Moore FA (2022). Nonocclusive mesenteric ischemia: a rare but lethal complication of enteral nutrition in critically ill patients. Nutr Clin Pract.

[10] Yu B, Ko RE, Yoo K, Gil E, Choi KJ, Park CM (202219). Non-occlusive mesenteric ischemia in critically ill patients. PLoS One.

[11] Paul M, Bougouin W, Legriel S (2020). Frequency, risk factors, and outcomes of non-occlusive mesenteric ischaemia after cardiac arrest. Resuscitation.

[12] Chiu HH, Chen CM, Lu YY, Lin JC, Mo LR (2005). Hepatic portal venous gas. Am J Surg.

[13] Yu H, Kirkpatrick ID (2023). An update on acute mesenteric ischemia. Can Assoc Radiol J.

[14] Auxiliadora-Martins M, Alkmin-Teixeira GC, Feres O, Martins-Filho OA, Basile-Filho A (2010). Fulminant nonocclusive mesenteric ischemia just after hip arthroplasty. Case Rep Med.

[15] Espiritu CR, Robinson MJ (1975). The clinical presentation of mesenteric vascular disease. South Med J.

[16] Flynn AD, Valentine JF (2016). Update on the diagnosis and management of colon ischemia. Curr Treat Options Gastroenterol.

[17] Takiguchi T, Arai M, Kim S (2021). Nonocclusive mesenteric ischemia associated with a hyperosmolar hyperglycemic state: hepatic portal venous gas as an indicator of mesenteric ischemia. Acute Med Surg.

[18] Adam N, Kandelman S, Mantz J, Chrétien F, Sharshar T (2013). Sepsis-induced brain dysfunction. Expert Rev Anti Infect Ther.

